# The level of actual functional fitness of men from different living environments in Poland and associations with health - a cross-sectional study

**DOI:** 10.1186/s12877-023-04577-8

**Published:** 2023-12-12

**Authors:** Antonina Kaczorowska, Anna Sebastjan, Małgorzata Kołodziej, Małgorzata Fortuna, Zofia Ignasiak

**Affiliations:** 1 Institute of Health Sciences, University of Opole, ul. Katowicka 68, Opole, 45-060 Poland; 2 Department of Biostructure, Wrocław University of Health and Sport Sciences, Wrocław, Poland; 3 Department of Biomechanics, Wrocław University of Health and Sport Sciences, Wrocław, Poland; 4 Faculty of Health and Physical Culture Sciences, The Witelon State University of Applied Sciences in Legnica, Legnica, Poland

**Keywords:** Aging, Physical fitness, Aged, Nursing homes, Housing for the elderly

## Abstract

**Background:**

The study aimed to assess the differences in functional fitness between older men living in social welfare homes (SWH) and men living in society.

**Methods:**

The study involved 474 men aged 60–84, including 134 men living in social welfare homes and 340 men living in community. The Senior Fitness Test was used to assess functional fitness. Body weight and height were measured. Moreover, data on age, education, taking up physical activity and place of residence were collected.

**Results:**

A significantly lower level of functional fitness of SWH residents was demonstrated compared to men living in the society (*p* < 0.001). A significant percentage of seniors living in SWH did not reach the normal range for the Polish population. Also, the level of education, physical activity and health self-assessment differentiated the institutionalized men from those living in the community.

**Conclusions:**

The place of residence is a factor that differentiates seniors’ functional fitness levels. The reduced fitness of social welfare home residents may also result from the low level of their daily physical activity, education and health. Introducing physical activity programs for elderly residents in social welfare homes seems reasonable.

**Trial registration:**

ISRCTN platform as 18,225,729; December 2020.

## Background

The ageing of the population is a long-term trend that began several decades ago in Europe and the world. This tendency is visible in the transformations of the population’s age structure. It is reflected in the growing share of the elderly, with a decreasing percentage of people aged up to 20 in the total population. It is predicted that by 2100 people aged 65 or older will represent 31.3% of the population of the European Union, compared with 20.8% in 2022 [1]. Also, in Poland, the ageing of society has been increasing in recent years. The COVID-19 pandemic hit the hardest among the elderly, among whom the death rate was the highest. In Europe, 2,077,938 people have died due to COVID-19 since the beginning of the pandemic, and 117,130 people in Poland [2]. Therefore, in the coming years, the dynamics of the ageing of society in Europe may slow down and transform to some extent. However, in the decades to come, the senior population will increase [1].

Physical fitness related to health and regular physical activity are critical factors in maintaining health-related well-being [3, 4]. It has been documented that improving health-related physical condition, including cardiovascular fitness, muscle strength and endurance, flexibility and body composition, is vital to improving health and quality of life at all ages [5–8]. Maintaining a satisfactory level of functional physical fitness through regular exercise is very beneficial in alleviating and preventing metabolic diseases [9–11] in cardiovascular and respiratory disorders [12, 13], in conditions of the osteoarticular system [14, 15] and the more and more often occurring sarcopenia and frailty syndrome [16, 17]. The American College of Sports Medicine and the American Heart Association published a recommendation clearly identifying the beneficial role of high-intensity exercise. Adults are encouraged to combine moderate and vigorous intensity exercise with meeting minimum physical activity recommendations [12]. A study by Wu et al. showed that high-intensity interval training induces favorable adaptations in the case of cardiopulmonary fitness, physical fitness and muscle strength in the elderly, which may help maintain aerobic performance and slow down the development of sarcopenia [8]. Physical activity not only reduces total morbidity and mortality in the elderly, but also improves functionality and reduces disability in terms of aerobic capacity, body composition, muscle mass, bone density, and articulatory ability [18]. Exercise-based interventions are an effective way to prevent falls in older people and improving functional efficiency [19]. American studies have proven that resistance exercises among people over 50 effectively induce strength gain and lean body mass gain. The more visible beneficial adaptive changes are closely related to the volume and intensity of the performed physical work. Progressive resistance exercises minimize degenerative muscle functions related to ageing [17]. Older people should be encouraged to break away from their daily sedentary lifestyle and avoid long periods of sitting [20]. Regular physical activity also increases resistance to infectious and viral diseases [5, 21].

A decrease in physical activity and exercise tolerance with age is physiological and inevitable, but the pace of this decrease varies among individuals [22]. Researchers report that the rate of changes is lower in the group of seniors, who engage in systematic physical activity and are characterized by an active family and social lifestyle [23, 24] and higher education [25, 26].

There is a lower percentage of men in the studied populations of seniors [7, 16, 20]. In Poland, in 2020, the percentage of men in the population of people over 60 was 48.9%. The percentage of feminization increases with age in the group of people at a senior age. Therefore, the percentage of surveyed women is greater than that of men. Perhaps it is a consequence of the excess mortality rate of men in Poland and Europe [27, 28], especially among those with the lowest level of education [29]. Based on the results of these studies, it seems essential to analyze the ageing process of men in terms of the factors that may affect the maintenance and duration of functional independence, health, and quality of life.

In recent years, significant changes in the structure of families have been observed. Multigenerational families are disappearing, and the elderly show more interest in staying in senior centers. Older people are most often placed in residential care homes due to age, illness and disability, because of which they are unable to function independently [30]. In turn, dependency in activities of daily living is associated with an increased risk of morbidity and mortality [31]. Therefore, in order for seniors in institutions to have a good quality of life and a long life, continuous monitoring of their physical condition and health status is essential. There is a lot of current research on the functional fitness and health status of older people living in the community [22, 24, 32–35] but much less research on institutionalized older people [36–39] and especially in Poland [16, 40, 41]. Furthermore, the associations of physical fitness with physical activity and health status among seniors living in social welfare homes and in the community have been poorly studied. Further research is needed to understand them better.

The study aimed to assess the level of functional physical fitness of men in southwest Poland living in social welfare homes and men living in society in terms of health.

## Methods

### Study design, setting and participants

The research was conducted in southwest Poland. The study design included an assessment of the functional fitness, health and physical activity of older men from different living environments and a comparison of the results obtained by the subjects in the Senior Fitness Test to norms developed for the Polish population. The study protocol was approved by the Senate Research Ethics Committee at the Wrocław University of Health and Sport Science (Approval Date: 2009) and complied with the ethical requirements for human experimentation following the Declaration of Helsinki. The study is part of an extensive research that was retrospectively registered on the ISRCTN platform as. 18,225,729. The STROBE (Strengthening the Reporting of Observational Studies in Epidemiology) guidelines were followed.

The study involved 474 men aged 60–84, including 134 men living in social welfare homes (SWH) and 340 men living in the community. The study involving SWH residents was carried out in 12 social welfare homes in southwest Poland. The management of all the centers agreed to the study. The comparison group consisted of men living independently from southwest Poland, participating in a multi-stage project involving the assessment of the physical and biological condition of elderly people in Poland between 2010 and 2016. All participants were white (Caucasian). SWH residents also previously lived in southwestern Poland.

The inclusion criteria for both groups included the age of 60–84, the ability to move independently and perform the Senior Fitness Test, no medical contraindications, and free, written consent to participate in the study. The exclusion criteria included cancer, acute trauma and infections, febrile states, recent myocardial infarction, other medical contraindications, and refusal to participate in the study.

Out of all 440 residents of the SWH, 225 were unable to perform the fitness test, 32 had medical contraindications, 35 did not meet the age criterion, and 14 did not consent to the tests. Hence, the final study group of SWH residents consisted of 134 people. All the participants living in the community volunteered for free tests thanks to media advertisements and invitations to centers associating the elderly. Out of 405 men, 65 did not meet the age criterion. Ultimately, the group of men living in the community amounted to 340 individuals. The study was carried out in the afternoon by the same researchers.

The participants were informed about the purpose and methods of the study as well as the procedures. All the individuals who declared participation in the study signed a document of free and informed consent.

### Data and measurements

Data on age, education, place of residence, physical activity and health self-assessment were collected based on a short questionnaire. Basic education included completed primary school or completion of at least three grades of primary school. Vocational education included the completion of a two-year vocational school. Secondary education included the completion of secondary school - a four-year high school or a five-year technical school. Higher education included the completion of a university degree. Respondents could choose between village, small and medium-sized towns (up to 100 000 inhabitants) and big cities (over 100 000 inhabitants) as their place of residence. Height and body weight were measured with an accuracy of 0.1 cm and 0.1 kg, respectively, using an electronic balance with an integrated SECA 764 digital stadiometer (Seca GmbH & Co. KG. Germany).

Functional fitness was assessed using the Senior Fitness Test [42]. This test is designed to assess the functional fitness of the elderly and consists of 6 trials. As not all social welfare home residents were able to perform the test to assess aerobic performance, the remaining 5 Senior Fitness Test trials were used to evaluate their functional fitness: a 30-second chair stand (lower body strength evaluation), arm curl (upper body strength evaluation), chair sit and reach (lower body flexibility evaluation), back scratch (upper body flexibility evaluation), and 8-foot up and go (assessment of walking speed and dynamic balance).

### Statistical analysis

For categorical data, the frequency of occurrence (%) was calculated. The results of all the measurements are presented as mean ± standard deviation (Mean ± SD). The normality of the distribution of continuous data was checked with the Shapiro-Wilk test, which confirmed the convergence with the normal distribution only in the groups taking into account the age of the subjects. The results of the functional fitness tests were compared to the norms for the elderly population in Poland developed by Ignasiak et al. [43]. For all the tests, the following categories of results were adopted: “Below the norm” – for results below the 25th percentile in the 30-Second Chair Stand Test, Arm Curl Test, Chair Sit and-Reach Test, Back Scratch Test, and for results above the 75th percentile in 8-Foot Up-and-Go Test. All the other values ​​were taken as the results of functional efficiency “within the norm”. The cut-off points for reduced functional fitness are shown in Table 1.

**Table 1:** Cut-off points for reduced functional fitness in men assessed using the Senior Fitness Test battery (according to Ignasiak et al. (2020))

Age (years)	60–64	65–69	70–74	75–79	80–84
30-Second Chair Stand Test (no. of reps)	< 13	< 13	< 12	< 12	< 11
Arm Curl Test (no. of reps)	< 17	< 17	< 16	< 14	< 13
Chair Sit and-Reach Test (cm)	< -2	< -4	< -7.5	< -8	< -9
Back Scratch Test (cm)	< -17	< -18	< -20	< -21	<-22
8-Foot Up-and-Go Test (s)	> 6.2	> 6.5	> 6.7	> 7.3	> 8.5

Note: The cut-off points were established based on the research results achieved among 291 men aged 60–64; 320 men aged 65–69; 260 men aged 70–74; 176 men aged 75–79; 82 men aged 80–84

The Mann-Whitney U test for continuous data (which were not normally distributed if the age of the respondents was not taken into account) and Pearson’s χ^2^ test for categorical data were used to assess the differences between the inhabitants of social welfare homes and men living in the community. The differences in functional fitness by age groups specified in Table 1 were checked by multi-dimensional (MANOVA) and one-dimensional (ANOVA) analyses from Tukey’s honestly significant difference test (HSD). The statistical significance of the results was accepted at *p* < 0.05.

## Results

The descriptive characteristics of the subjects are presented in Table 2. Apart from their age, all the analyzed parameters significantly differentiated both groups of men (*p* < 0.05). Compared to males living in the community, men in SWH were characterized by lower body weight, height and body mass index (BMI). Over 70% of them had primary and vocational education and most often came from smaller and medium-sized towns. In the case of participants living independently, as many as 92% had at least secondary education. Social welfare home residents declared a lack of physical activity more often than men from the comparison group. One third of the men in SWH assessed their health as bad, whereas the rest declared being in good health. Among men living in the community, the percentage of individuals declaring good and very good health exceeded 97%.

The results of functional fitness tests indicated that the majority of men living in SWH have significantly reduced physical fitness, and depending on the type of test, from 53% to even 91% of them, do not achieve the results typical for the population of elderly people in Poland (Ignasiak et al. 2020). Among the community-dwelling seniors, the tests assessing the flexibility of the lower and upper body were the worst: 19.7% and 16.2% of the studied men did not reach the Polish norms (Table 2).

**Table 2:** Descriptive characteristics of study participants, mean ± standard deviation and frequency of occurrence (%), U Mann-Whitney test and χ^2^ test

	Men living in Social Welfare Homes-SWH (n = 134)	Men living in the community-no SWH (n = 340)	*p*
**Age (years)**	69.7 ± 7.3	68.6 ± 5.2	0.518
**Height (cm)**	167.9 ± 7.9	172.4 ± 5.7	< 0.001
**Weight (kg)**	74.4 ± 14.8	86 ± 12.5	< 0.001
**BMI (kg/m** ^**2**^ **)**	26.4 ± 4.8	28.9 ± 3.7	< 0.001
**Education**			< 0.001
basic	38.8%	2.1%	
vocational	31.3%	5.9%	
secondary	24.6%	38.2%	
higher	5.2%	53.8%	
**Place of residence**			< 0.001
village	8.2%	15.0%	
small and medium-sized town	72.4%	46.5%	
big city	19.4%	38.5%	
**Taking up physical activity**			< 0.001
no	40.3%	12.7%	
occasionally (sometimes)	37.3%	28.2%	
yes	22.4%	59.1%	
**Subjective health condition assessment**			
bad	30.6%	2.6%	
good	64.9%	90.0%	< 0.001
very good	4.5%	7.4%	
**30-Second Chair Stand Test (no. of reps)**	10.1 ± 4.3	18.9 ± 5.3	< 0.001
below the norm for Polish seniors	71.6%	5.9%	
**Arm Curl Test (no. of reps)**	14.4 ± 4.3	23.5 ± 5.2	< 0.001
below the norm for Polish seniors	53.0%	5.3%	
**Chair Sit and-Reach Test (cm)**	-11.7 ± 12.8	2.3 ± 9.7	< 0.001
below the norm for Polish seniors	62.7%	19.7%	
**Back Scratch Test (cm)**	-23.6 ± 14.9	-8.4 ± 10.6	< 0.001
below the norm for Polish seniors	57.9%	16.2%	
**8-Foot Up-and-Go Test (s)**	14.4 ± 7.7	5.1 ± 0.9	< 0.001
below the norm for Polish seniors	91.0%	3.5%	

BMI – body mass index

The MANOVA results indicated that the effect differentiating functional fitness parameters was not only the place of residence (*p* < 0.001) but also age (*p* < 0.001) and the interaction of age with the place of residence (p = 0.036). Detailed results of comparisons in individual age groups with marked significant differences between social welfare home residents and men living in the community are presented in Table 3. In each age group, all fitness parameters of social welfare home residents were significantly reduced compared to other men. Moreover, lower anthropometric parameters were observed in social welfare home residents in the two youngest groups (60–64 years and 65–69 years).

In the 30-Second Chair Stand Test and Arm Curl Test, the men in social welfare homes performed approximately 40–60% fewer repetitions than the men in the comparison group. Even more significant differences were observed in the flexibility test, especially in the younger groups (< 75 years), in which the average flexibility scores of the residents did not reach the positive values ​​characteristic for the comparison groups. The results of the 8-Foot Up-and-Go test indicated that, depending on the age, the walking speed of men in SWH was 1.5 to 2 times lower than that of men living independently (Table 3).

**Fig. 1:**
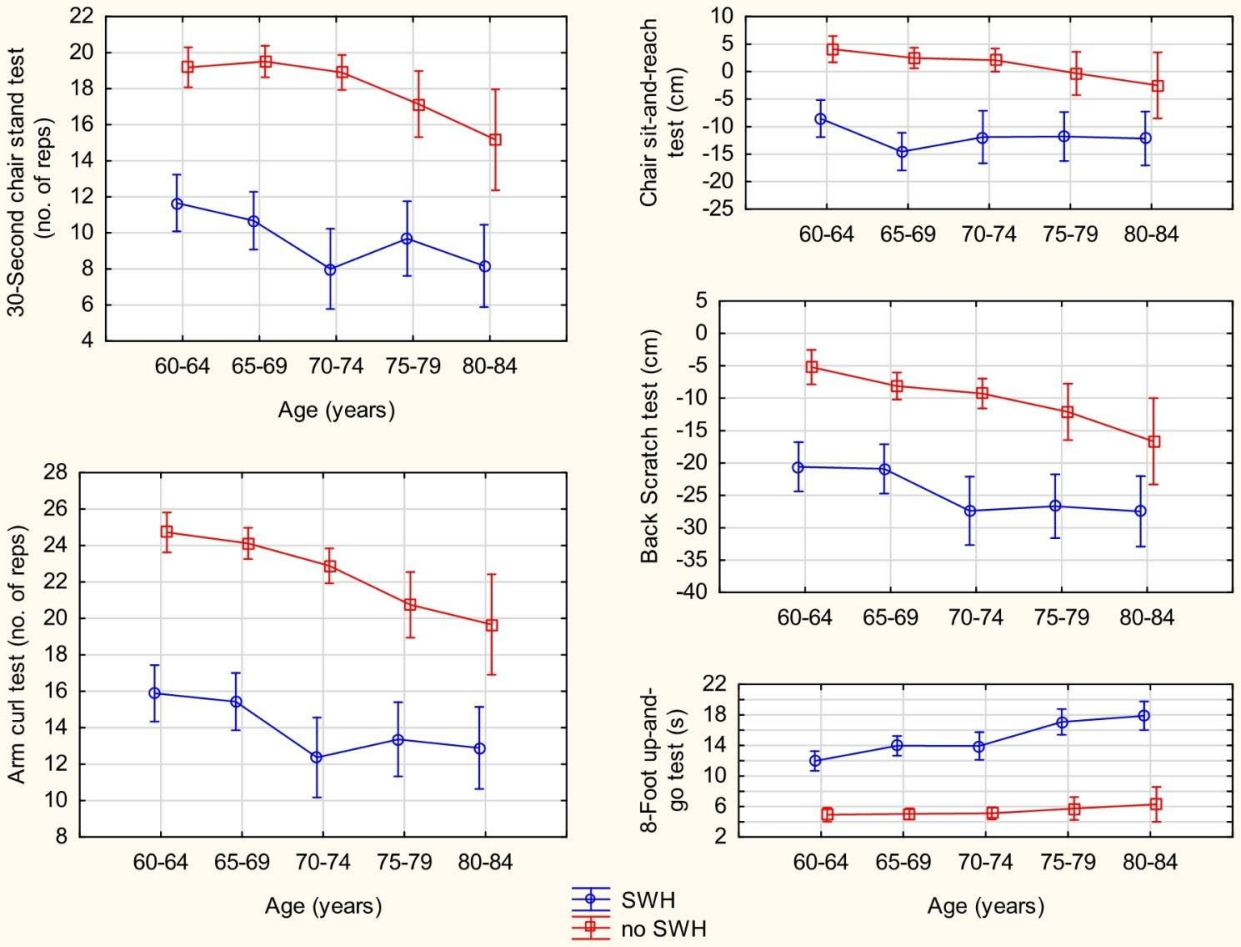
Differences in functional fitness between men living in social welfare homes (SWH) and men living in the community (no SWH), taking into account the age of the study participants. Points with vertical lines represent the mean with 95% confidence intervals

The size of the differences between the age groups, which may characterize the dynamics of changes in functional fitness depending on the place of residence cross-sectionally, is presented in Fig. 1. As expected, functional fitness decreases with age, regardless of the place of residence. Still, in the age range of 60–75, the rate of changes in the results of individual fitness tests with age is greater in men living in social welfare homes. In all the tests, the differences between the two cohorts were so significant that even 60-64-year-old social welfare home residents did not achieve the functional fitness of men 20 years older than those living in the community (Fig. 1).

**Table 3:** Differences between men living in social welfare homes (SWH) and community-dwelling men (no SWH), mean ± standard deviation

	60–64 Years		65–69 Years		70–74 Years		75–79 Years		80–84 Years	
	SWH N = 38	no SWH N = 76	SWH N = 37	no SWH N = 124	SWH N = 19	no SWH N = 100	SWH N = 22	no SWH N = 28	SWH N = 18	no SWH N = 12
Age (years)	**61.5 ± 1.6**	**62.2 ± 1.4**	66.6 ± 1.4	67.1 ± 1.5	72.2 ± 1.3	71.6 ± 1.5	76.7 ± 1.5	77.0 ± 1.3	81.8 ± 1.4	81.8 ± 1.2
Height (cm)	**169.3 ± 7.5**	**173.8 ± 5.9**	**169.9 ± 8.3**	**173.5 ± 5.3**	168.8 ± 8.0	171.2 ± 5.6	**163.4 ± 7.2**	**170.5 ± 5.3**	165.1 ± 6.4	167.6 ± 6.3
Weight (kg)	**72.9 ± 16.8**	**87.5 ± 12.0**	**72.1 ± 12.8**	**88.1 ± 12.7**	**78.3 ± 14.0**	**85 ± 12.6**	77.1 ± 16.4	79.6 ± 8.9	75.3 ± 12.8	79.3 ± 12.8
BMI (kg/m^2^)	**25.3 ± 4.9**	**28.9 ± 3.5**	**24.9 ± 4.1**	**29.2 ± 3.7**	27.3 ± 3.6	29.0 ± 3.9	28.9 ± 5.3	27.4 ± 2.9	27.7 ± 4.7	28.1 ± 3.2
30-Second chair stand test (no. of reps)	**11.7 ± 4.3**	**19.2 ± 4.8**	**10.7 ± 4.3**	**19.5 ± 5.7**	**8.0 ± 4.0**	**18.9 ± 4.9**	**9.7 ± 3.7**	**17.1 ± 5.2**	**8.2 ± 4.2**	**15.2 ± 4.5**
Arm curl test (no. of reps)	**15.9 ± 4.0**	**24.7 ± 4.4**	**15.4 ± 4.1**	**24.1 ± 5.5**	**12.4 ± 3.5**	**22.9 ± 5.3**	**13.4 ± 4.4**	**20.8 ± 4.6**	**12.9 ± 4.7**	**19.7 ± 4.0**
Chair sit-and-reach test (cm)	**-8.5 ± 15.1**	**4.1 ± 8.8**	**-14.6 ± 12.0**	**2.5 ± 9.6**	**-11.9 ± 10.5**	**2.1 ± 10.1**	**-11.8 ± 11.2**	**-0.3 ± 9.3**	**-12.2 ± 12.6**	**-2.5 ± 10.8**
Back Scratch Test (cm)	**-20.6 ± 15.9**	**-5.2 ± 9.2**	**-20.9 ± 14.9**	**-8.1 ± 10.7**	**-27.4 ± 15.9**	**-9.3 ± 10.2**	**-26.7 ± 13.1**	**-12.1 ± 11.3**	**-27.5 ± 13.0**	**-16.7 ± 12.3**
8-Foot up-and-go test (s)	**12.0 ± 5.2**	**4.9 ± 0.7**	**14 ± 7.8**	**5.0 ± 0.8**	**13.9 ± 5.3**	**5.1 ± 0.9**	**17.1 ± 10.3**	**5.7 ± 0.9**	**17.9 ± 9.0**	**6.3 ± 1.1**

Statistically significant differences at *p* < 0.05 are marked in bold.

## Discussion

### Key results and interpretation

The study aimed to assess the functional fitness of elderly people living in different living conditions (social welfare homes and living in the community) in terms of health. The anthropometric carried out measurements show that men living in the community are taller and heavier compared to men in SWH, and their BMI indicates greater overweight. The analysis of the primary socio-economic factors shows that men’s education level was different. Men in social welfare homes are characterized by a significantly lower level of education than men living in the community, among whom higher and secondary education dominates. The second factor – the size of the place of residence also significantly differentiates the two groups of men. Most often, men from small and large cities live in social welfare homes. This may be due to a greater sense of loneliness and less resourcefulness in everyday life. The infrastructure of cities often creates many difficulties and barriers limiting independent functioning. This fact is related to undertaking systematic physical activity. Almost 80% of men in SWH avoid regular physical exercise, while in the case of men living independently the situation is the opposite. As a result, functional physical fitness is significantly higher among men living in the community (*p* < 0.001). As far as Polish norms are concerned, a high percentage of community-dwelling men fall within the range of the norm developed for Polish males [43].

Men living in social welfare homes have a very low level of functional fitness and do not reach the standard value in a very high percentage. The results of the gait speed and dynamic balance test are particularly worrying. Over 90% of the surveyed males are not within the norm, indicating a high risk of falls and serious consequences, such as tissue contusions, joint dislocations or bone fractures [44–47].

Other researchers confirm the low fitness levels of SWH residents. Kaczorowska et al. studied older women aged 65–79 years from SWH and living in the community using the Senior Fitness Test. Scores on all attempts of the test were found to be significantly lower in the group of female SWH residents compared to seniors living in the community. The weakest attempts in the SWH group were 8-foot up and go and 30-second chair, in which, respectively, 87.3% and 74.6% of the seniors tested did not reach the norms developed for the Polish population [40]. In another Polish study of the functional fitness of older women from SWH, the weakest tests were 8-foot up and go and 6-minute walk, in which only a few percent of the seniors tested met Polish norms [48]. The Brazilian study included older women living in the community and receiving day care. The institutionalized women showed lower physical fitness, as assessed by the Senior Fitness Test, compared to women living in the community. In addition, they showed a clear trend of decreasing fitness levels over time [49].

The low level of functional fitness of social welfare home residents may be related to the limitation of daily activities. Among the surveyed seniors, little more than 20% of residents in SWH undertake systematic physical activity. Social welfare homes in Poland operate on the basis of the Regulation of the Minister of Labor and Social Policy on social welfare homes of 2012, with the changes introduced in 2017 and the final amendments in 2018 [50]. Apart from services in the field of living needs and care services, a social welfare home should also provide therapeutic services, which include, among other things, enabling participation in occupational therapy, increasing fitness, and activating the inhabitants. The employment rate of employees of therapeutic and care teams is the lowest in social welfare homes for the elderly. It amounts to no less than 0.4 per one inhabitant of a social welfare home, compared to 0.5 or 0.6 per one inhabitant of a social welfare home in other types of SWH (for example for the chronically mentally ill, for people with physical disabilities). The regulation also does not specify how many occupational therapists and physiotherapists should be in a therapeutic and care team [50]. More often than not, the number of therapists employed in a social welfare home is too small, considering the number of residents. Physiotherapists also do not always encourage residents to exercise sufficiently, focusing on anti-inflammatory and analgesic physical treatments. Moreover, residents of a social welfare home very often do not agree to participate in therapeutic classes. Some people consciously refuse to participate in them. It is imperative to activate the residents of SWH and skillfully encourage them to participate in therapeutic activities. That is why the selection of therapeutic staff is so important. Physiotherapists and occupational therapists should be able to activate seniors to participate in the classes. Physical activity of older SWH residents increases their functional fitness and independence and improves their quality of life, but it is also essential for economic reasons. Thus, the more active a senior is and the more they participate in physical activities, the healthier they are, the less self-service they require, and the lower their financial outlays. Therefore, occupational therapy classes and physical rehabilitation of seniors living in social welfare homes should be intensified. This is confirmed by studies by other authors. Research by Peng et al. has shown that a group exercise program is helpful in improving the physical fitness, fragility and health of elderly nursing home residents [36].

The problem of too little activation of seniors living in social welfare homes is also noticed in other European countries. A study in German social welfare homes showed that forms of exercise were not adequately communicated to residents, and less than half of the elderly residents participated in them. A wide range of forms of exercise was widely available, but they were rarely tailored to the needs of seniors [51]. A study in Great Britain showed that the level of physical activity of care home residents was very low − 79% of the respondents spent their day sitting [37]. Elderly residents of care homes in Spain also had a low level of daily physical activity as measured by an accelerometer [38].

However, based on our own research, we cannot unequivocally conclude that the reason for poor physical fitness is low levels of physical activity. The opposite may be true - it is poor health that hinders physical activity and lowers the level of functional fitness. SWH residents rated their health significantly worse compared to independent living residents. Almost one third of the examined SWH residents rated their health as bad. In Poland, the right to live in a SWH is granted to persons who cannot function independently in daily life due to age, illness or disability [30]. This is why the functional fitness of the examined SWH residents may be so low. These observations indicate that there is a feedback loop between functional fitness, physical activity and health status. And it is difficult to determine which factor is more decisive. As physical fitness promotes health, independence and quality of life at all ages, further research on its relationship with health status and physical activity levels is needed.

The level of quality of life and physical fitness is determined by many factors. One of them is education. A study of Polish older men found an independent effect of age and education on the severity of psychological, sexual and somato-vegetative symptoms. The better the education of Polish men was, the less severe the aging symptoms of men were, regardless of their age [25]. Other Polish authors confirmed that age and education were most strongly associated with the severity of almost all symptoms of ageing in adult men [26]. Researchers emphasize that the level of awareness associated with a healthy lifestyle is related to economic status and the level of education [52]. Other authors have shown a relationship between the level of education and the course of the ageing process and physical activity [53]. In Australian studies, people with lower education less frequently engaged in moderate and intense physical activity [54]. This is probably why the lower level of education among social welfare home residents in our study translated into a low level of physical activity, physical fitness and subjective health assessment. In addition, seniors living in the community with a higher education are also taller than SWH residents. This result confirms that there are clear social gradients in terms of body height in Poland. Men with better SES are significantly taller than their peers with lower SES [55].

Our study also showed that the decline rate in the level of functional fitness increases with age. Based on cross-sectional analyses in 5 age groups, we observed a greater reduction in fitness among SWH residents, especially in the age range of 60–75, compared to their peers living in the community. These results may suggest that functional changes in the ageing process may be faster in the case of individuals in care institutions. The research of other authors confirms this. Sousa and Mendes [56] showed a quicker regression in physical fitness among elderly women living in social welfare homes than among people living in the community in the same age range, suggesting that institutionalization is associated with a marked decline in physical activity. On the other hand, the admission of seniors to SWH at such a relatively young age (60–75 years) may be due to the deterioration of their health. And that is why their functional capacity is so low.

The low results obtained by the study participants living in social welfare homes in our study in relation to Polish reference norms indicate an urgent need to activate institutionalized seniors. When designing and implementing physical activity programs for seniors living in social welfare homes, the factors discussed in this study, such as age, anthropometric features, physical activity and health status, should be considered. However, the physical fitness of SWH residents can be always negatively different compared to those whose health condition does not require placement in SWH.

### The study’s strengths

Our study has several strong points. We examined the optimal number of men from the region of southwest Poland. We carried out measurements of the actual functional fitness. They provide more reliable assessments of the level of physical fitness in older men (the evaluation of the strength of the muscles of the upper and lower body, flexibility characterizing the range of motion in the main joints of the musculoskeletal system, and the speed of gait and dynamic balance) than survey studies. The Rikli & Jones (2002) test we used was developed for the elderly and is safe and reliable. A survey is commonly used in screening and epidemiological studies. Survey studies are probably more convenient and more manageable. Still, at the same time, they are burdened to a greater extent by the examined person’s subjectivity, mood, and state of cognitive functions.

### Limitations of the study

We did not avoid certain limitations in our study. These include the cross-sectional nature of research that makes it impossible to track the dynamics of changes in involution processes. The studied population lived in a selected region of Poland, so the obtained results cannot be applied to the entire Polish population, given the possibility of differences in the level of socio-economic factors (SES). Another limitation was voluntary participation in the study. It can be assumed that more physically and socially active people applied to participate.

We also did not ask how many SWH residents had children and whether they lived in villages or cities at an early age. This information could also affect the results. We will take them into account in the next surveys.

## Conclusions

Place of residence is a factor that differentiates seniors’ functional fitness levels. The reduced fitness of social welfare home residents may result from the low level of their physical activity, education and poor health.

It seems reasonable to introduce physical activity programs for elderly residents of social welfare homes to limit the decline in physical fitness and independence in their everyday life.

## Data Availability

The data presented in this study are available on request from the corresponding author.

## Declarations

### Guideline (Accordance)

The study was complied with the ethical requirements for human experimentation following the Declaration of Helsinki.
